# Genome-Wide Analysis of the TORC1 and Osmotic Stress Signaling Network in *Saccharomyces cerevisiae*

**DOI:** 10.1534/g3.115.025882

**Published:** 2015-12-16

**Authors:** Jeremy Worley, Arron Sullivan, Xiangxia Luo, Matthew E. Kaplan, Andrew P. Capaldi

**Affiliations:** * Department of Molecular and Cellular Biology, University of Arizona, Tucson, Arizona, 85721-0206; † Functional Genomics Core Facility, University of Arizona, Tucson, Arizona, 85721-0206

**Keywords:** TORC1, osmotic stress, yeast knock-out collection, high-throughput screen

## Abstract

The Target of Rapamycin kinase Complex I (TORC1) is a master regulator of cell growth and metabolism in eukaryotes. Studies in yeast and human cells have shown that nitrogen/amino acid starvation signals act through Npr2/Npr3 and the small GTPases Gtr1/Gtr2 (Rags in humans) to inhibit TORC1. However, it is unclear how other stress and starvation stimuli inhibit TORC1, and/or act in parallel with the TORC1 pathway, to control cell growth. To help answer these questions, we developed a novel automated pipeline and used it to measure the expression of a TORC1-dependent ribosome biogenesis gene (*NSR1*) during osmotic stress in 4700 *Saccharomyces cerevisiae* strains from the yeast knock-out collection. This led to the identification of 440 strains with significant and reproducible defects in NSR1 repression. The cell growth control and stress response proteins deleted in these strains form a highly connected network, including 56 proteins involved in vesicle trafficking and vacuolar function; 53 proteins that act downstream of TORC1 according to a rapamycin assay—including components of the HDAC Rpd3L, Elongator, and the INO80, CAF-1 and SWI/SNF chromatin remodeling complexes; over 100 proteins involved in signaling and metabolism; and 17 proteins that directly interact with TORC1. These data provide an important resource for labs studying cell growth control and stress signaling, and demonstrate the utility of our new, and easily adaptable, method for mapping gene regulatory networks.

The Target of Rapamycin (TOR) kinases are conserved across eukaryotes, where they act as master regulators of cell growth and metabolism (Loewith and Hall 2011; Laplante and Sabatini 2012). In line with their central role in cell signaling, TOR kinases respond to an enormous array of stimuli and control the activity of hundreds of proteins—functions that are supported in part by their recruitment into two distinct complexes: TOR Complex 1 (TORC1), and TOR Complex 2 (TORC2) (Barbet *et al.* 1996; Kim *et al.* 2002; Loewith *et al.* 2002; Urban *et al.* 2007; Huber *et al.* 2009; Soulard *et al.* 2010; Hsu *et al.* 2011). TORC1, unlike TORC2, is rapamycin sensitive, and in *Saccharomyces cerevisiae* is made up of the TOR kinase Tor1 (and, in its absence, the homolog Tor2), the key regulator Kog1, and two poorly characterized proteins, Lst8 and Tco89 (Heitman *et al.* 1991; Loewith *et al.* 2002; Reinke *et al.* 2004).

In the presence of adequate nutrients, TORC1 drives growth by activating multiple steps in protein and ribosome synthesis. First, TORC1 directly phosphorylates and activates the transcription factor Sfp1, and the AGC kinase Sch9 (Urban *et al.* 2007; Lempiainen *et al.* 2009). Sch9, in turn, then phosphorylates and blocks the activity of the transcriptional repressors Dot6, Tod6, and Stb3, leaving Sfp1 to promote the high level expression of 400 genes involved in ribosome biogenesis (Ribi), and translation (Jorgensen *et al.* 2004; Marion *et al.* 2004; Liko *et al.* 2007; Lippman and Broach 2009; Huber *et al.* 2011). Second, TORC1 acts in cooperation with Yak1 and the cAMP dependent protein kinase (PKA) pathway, to promote the activity of Fhl1, and upregulate expression of the ribosome protein (RP) genes (Martin *et al.* 2004; Schawalder *et al.* 2004; Wade *et al.* 2004). Third, TORC1-Sch9 phosphorylates and regulates the kinase Maf1, and other factors, to activate Pol I and Pol III, and thus rRNA and tRNA synthesis (Upadhya *et al.* 2002; Huber *et al.* 2009; Lee *et al.* 2009). Finally, TORC1 promotes translation, in part by blocking phosphorylation of eIF2 (Barbet *et al.* 1996; Loewith and Hall 2011).

In contrast, when cells are starved for energy, amino acids, or nitrogen, or exposed to noxious stress, TORC1 signaling is inhibited, leading to downregulation of Ribi and RP gene expression, rRNA and tRNA synthesis, and consequently cell growth (Powers and Walter 1999; Gasch *et al.* 2000; Urban *et al.* 2007; Brauer *et al.* 2008). In particular, dephosphorylation of Dot6, Tod6, and Stb3 triggers recruitment of the Class I histone deacetylase Rpd3L to the Ribi and RP genes, leading to a rapid decrease in gene expression levels (Alejandro-Osorio *et al.* 2009; Lippman and Broach 2009; Huber *et al.* 2011).

The mechanisms underlying TORC1 inhibition in nitrogen and amino acid starvation conditions are starting to come into focus. Specifically, it is now clear that nitrogen and amino acid starvation trigger activation of the GAP Npr2-Npr3-Iml1 SEAC subcomplex, SEACIT, and this in turn alters the GTP binding state of the small GTPases, Gtr1/Gtr2 (Kim *et al.* 2008; Sancak *et al.* 2008; Binda *et al.* 2009; Neklesa and Davis 2009; Panchaud *et al.* 2013). Gtr1/Gtr2 then bind TORC1 on the vacuolar membrane, and inhibit TORC1-dependent phosphorylation of Sfp1 and Sch9 (Urban *et al.* 2007; Binda *et al.* 2009; Lempiainen *et al.* 2009; Panchaud *et al.* 2013). At the same time, an interaction between Gtr1/Gtr2, the small GTPase Rho1, and TORC1 promotes release of Tap42 from the TOR complex, triggering Tap42-PP2A-dependent reprogramming of nitrogen and amino acid metabolism (Cardenas *et al.* 1999; Duvel *et al.* 2003; Yan *et al.* 2006; Yan *et al.* 2012). At least in humans, Gtr1/Gtr2 signaling also depends on interactions with the vacuolar ATPase (V-ATPase) and amino acid transporters on the vacuolar membrane (Zoncu *et al.* 2011; Wang *et al.* 2015).

Outside of nitrogen and amino acid starvation conditions, however, very little is known about TORC1, and TORC1 pathway, regulation. Npr2/Npr3, Gtr1/Gtr2, and Rho1 play little-to-no role in transmitting glucose starvation, osmotic stress, heat stress and oxidative stress signals to TORC1-Sch9 (Binda *et al.* 2009; Hughes Hallett *et al.* 2014). Instead, the AMP-activated protein kinase Snf1 partially inhibits TORC1, and/or TORC1-Sch9, signaling during glucose/energy starvation, while the MAPK Hog1 plays a small role in regulating TORC1, and/or TORC1-Sch9, signaling in osmotic stress (Hughes Hallett *et al.* 2014). It is also known that TORC1 binds to stress granules during heat shock, but this interaction is not required for the initial stages of TORC1 inhibition (Takahara and Maeda 2012). Thus, most of the proteins and pathways that regulate TORC1 and/or TORC1-Sch9 signaling in noxious stress and energy starvation remain to be identified.

It is also unclear how the TORC1 pathway cooperates with other signaling pathways to regulate cell growth. Numerous studies have shown that the ras/PKA pathway regulates expression of the cell growth genes in glucose, primarily by acting in parallel with Sch9 to phosphorylate and regulate Sfp1 and Dot6/Tod6 (Jorgensen *et al.* 2004; Marion *et al.* 2004; Martin *et al.* 2004; Zurita-Martinez and Cardenas 2005; Slattery *et al.* 2008; Lippman and Broach 2009). It is also known that the inositol kinases Vip1 and Kcs1, and the inositol pyrophosphates they produce, act in parallel with TORC1 to regulate Rpd3L, and thus the Ribi and RP genes, during stress (Worley *et al.* 2013). However, it is unclear how Kcs1 and Vip1 are regulated and if/how other pathways cooperate with TORC1 to control cell growth.

Therefore, to push our understanding of TORC1 signaling and cell growth control forward, we carried out a screen to identify proteins that are required for the downregulation of Ribi gene expression in osmotic stress. Similar screens have been carried out previously to identify proteins involved in the Unfolded Protein Response (UPR), Heat shock factor 1 (Hsf1) response (in log growth conditions), and the amino acid starvation response—in each case using a GFP reporter placed under a relevant promoter (Jonikas *et al.* 2009; Neklesa and Davis 2009; Brandman *et al.* 2012). However, a GFP reporter cannot easily be used to study cell growth control since Ribi and RP genes are only transiently downregulated during stress, leading to relatively small (twofold) changes in Ribi and ribosome protein levels (Gasch *et al.* 2000; Lee *et al.* 2011). To get around this problem, we developed a novel automated pipeline that directly measures mRNA levels at the peak of the osmotic stress response (a 32-fold change in gene expression), and used it to measure Ribi gene expression in 4700 strains from the yeast knock-out (YKO) collection (Winzeler *et al.* 1999). This led to the identification of 440 strains with a reproducible and highly significant (*P* < 0.001) defect in Ribi gene repression during stress. We then went on to show that 53 of these strains also have a significant defect in the response to rapamycin, and are therefore missing genes that act downstream of TORC1.

Among the genes that act downstream of TORC1, we find numerous factors involved in transcription and chromatin remodeling including six subunits of Rpd3L, three subunits of the Elongator complex, three histone proteins, two histone demethylases, and components of the SWI/SNF, INO80 and CAF-1 chromatin remodeling complexes. We also identified 21 ribosome proteins and translation factors in the screen, nine of which act downstream of TORC1. Other genes in the growth control network have a wide variety of functions, but include 56 proteins involved in vacuolar function and vesicle transport, including 10 components of the V-ATPase, as well as five kinases, five methyltransferases, and nine membrane transporters. Finally, 17 genes in the network physically interact with TORC1, suggesting that we have identified numerous direct regulators and effectors of TORC1 signaling.

Overall, the data presented here provide a valuable resource for labs studying TORC1 signaling, cell growth control, or the environmental stress response, and demonstrate the utility of our novel and easily adaptable method for mapping gene regulatory networks in yeast and other organisms.

## Materials and Methods

### Automated pipeline

Inoculation, growth, treatment, and RNA isolation steps were performed on a Biomek FX liquid handling robot (Beckman Coulter) equipped an integrated plate hotel (Cytomat) and shaking incubator (Liconic). All 96-well plates were labeled with barcodes, and loaded onto the Biomek using a barcode scanner, to ensure that the plates remained in order and maintained their original orientation. OD_600_ measurements were taken with a plate reader (BioTek Synergy 2) in sterile 96-well plates (Greiner Bio-One) at 30°. Detailed descriptions of the protocols run on the Biomek are provided in Supporting Information, File S1.

### Cell growth and stress treatment

YKO collection strains were pinned onto YEPD agar plates using a Singer ROTOR robot and grown for 2 d at 30°. The yeast were then pinned from the agar plates into 96-well plates containing 100 μl of YEPD per well and grown for 18–22 hr at 30°. The overnight cultures were then used to inoculate 2.2 ml deep-well plates (VWR), containing 550 μl of YEPD, and one sterile 3.2-mm stainless steel mixing bead per well, to an OD_600_ of 0.05, and loaded into the Liconic Incubator (shaking at 1200 rpm and 30°). Once the median OD_600_ of a plate reached 0.60 (no wells reached an OD_600_ of > 0.8), 150 μl of each culture was transferred to a 2.2 ml 96-well plate containing 850 μl of RNAse Inactivation Buffer per well (RI Buffer; 4 M ammonium sulfate, 100 mM MES buffer, and 20 mM EDTA, pH 4.6), and mixed thoroughly by pipetting; 100 μl of 1.875 M KCl, or 1 μg/ml rapamycin in 30° YEPD was then added to each remaining culture (yielding final concentration of 0.375 M KCl or 200 ng/ml rapamycin), and the plate was returned to the incubator for 19 min (shaking at 1200 rpm and 30°). The plate was then moved back to the deck of the robot, and 150 μl of culture removed from each well and added to RI Buffer as described above. The plates containing RI Buffer and yeast were then stored at –20°.

### RNA purification

Plates containing cells in RI Buffer were defrosted by centrifugation (25 min at 3000 rpm at room temperature), and the supernatant removed from each well. The pelleted cells were then resuspended in 400 μl lysis buffer (4 M guanidine thiocyanate, 25 mM Na citrate, 0.5% N-lauryl sarcosine), and transferred to a 700 μl 96-well plate (Griener) containing 300 μl of zirconia/silica beads per well. The plates were then sealed with sterile foil and shaken for 5 min on a mini-Beadbeater-96 (Biospec). After a second round of centrifugation (25 min at 3000 rpm at 4°), the plates were loaded into the Biomek, where 100 μl of lysate was transferred to a sterile 96-well PCR plate (Thermo Scientific). At this point, 70 μl of isopropanol was added to each lysate and mixed for 1 min before adding 20 μl of MagMax binding beads (50% slurry in binding buffer; Ambion) to each well. The isopropanol, lysate, and bead mix was then mixed for 7 min to ensure all of the RNA in the sample bound to the beads, the plate was moved to a magnetic stand-96 (Ambion) for 5 min, and the liquid removed from each well. The beads were then washed with 150 μl of Wash Buffer 1 (1.7 M guanidine thiocyanate, 0.17% N-lauryl sarcosine, 33% isopropanol, 33 mM Na citrate, pH 7.0) for 5 min, followed by 150 μl of Wash Buffer 2 (2 M KCl, 80% ethanol, 2 mM Tris, pH 7.0) for 5 min. The DNA in each sample was then cleaved by treatment with Turbo DNAse (0.25 μl of 2 U/µl stock in 50 μl DNAse buffer from Life Technologies) for 25 min at room temperature. The RNA was then bound to the magnetic beads again by adding 100 μl of 1.5x Wash Buffer 1, and incubating for 5 min, the beads washed two more times with Wash Buffer 2 (5 min each), and dried for 10 min at room temperature. Finally, the purified RNA was eluted by mixing the beads with 30 μl of 55° elution buffer (1 mM sterile-filtered RNAse-free Tris pH 8.0) for 5 min, the plate was then returned to magnetic stand (to remove the beads), and the eluate transferred to a sterile PCR plate and stored at –80°.

### qPCR

One-step qRT-PCR reactions were performed using 5 μl RNA, TaqMan probes/primers from Lifetech (used at the recommended concentrations; probe and primer sequences not provided by company), and 5 μl PerfeCTa qPCR ToughMix, Low ROX (Quanta) in a 96-well PCR plate using an Agilent Stratagene Mx3005p cycler. One TaqMan probe bound to the reporter gene NSR1 (labeled with FAM dye), and the other bound to a control gene, PEX6 or NTF2 (labeled with JOE dye). The ROX normalized data from each plate was then analyzed using the Stratagene MxPro software and fluorescence thresholds (dRn) of 0.120 for FAM (NSR1) and 0.60 for JOE (PEX6). Samples that passed the FAM or JOE threshold after >28 cycles were discarded. This filtering caused us to drop data from about 200 strains in the YKO library; most of these strains grew very poorly in the 96-well plates, leading to a low RNA yield.

### qPCR normalization

To calculate the normalized NSR1/PEX6 and NSR1/NTF2 ratios, the FAM minus JOE (F–J) value was calculated for every well. The machine learning module, scikit-learn, in Python was then used to calculate the average F–J value for two populations on each plate—strains with expression defects, and strains without expression defects. This was done using Gaussian Mixture Models in the ‘scikit.mixture’ package with a covariance class of type ‘full’ for two-component analysis (http://scikit-learn.org/stable/modules/mixture.html#selecting-the-number-of-components-in-a-classical-gmm). The average F–J value for the strains without an expression defect was then subtracted from the F–J value of the entire plate, setting the mean of the plate (minus the outliers) to 0.0. All values were then multiplied by –1 so that higher RNA concentrations give higher NSR1/PEX6 ratios. This normalization had little impact on the list of strains that we identified as outliers in the screen but adjusts for the 0.3–0.6 cycle variation in the average NSR1/PEX6 ratio that we observe in separate runs on the qPCR machine.

### Application of the method to other organisms and problems

The automated pipeline described here can (in theory) be used to study a wide variety of organisms/cell types. In most cases, this will require knocking down protein targets using siRNA or CRISPRi prior to stress treatment. The lysis step will also have to be optimized for each cell type. However, for organisms that do not have a cell wall it should be possible to perform chemical lysis on the deck of the robot and proceed directly to the RNA purification step. Finally, the primer sets used in the qPCR step will have to be optimized for each pathway and organism. The method described here could also be used to study RNA decay if a transcription inhibitor is added to the cells just prior to stress treatment.

### Network reconstruction

Interactions between the top 440 genes/proteins in our screen were mapped using the protein–protein interaction data from BioGRID (version 3.4.125). TORC1 (Tor1, Kog1, Lst8, Tco89) was also added to our model as one merged node for reference. 275 proteins, including TORC1, form the major network, while 160 genes have no connection to any other of the 440 proteins identified in the screen. Note that the HSP70 family chaperones Ssa1 and Ssb1, and the RNA binding protein Slf1 were removed from the set (along with any proteins that only interact with them) in Figure 6 to eliminate nonspecific interactions (leaving 236 genes).

The interactions within the osmotic stress response network were mapped and clustered using Cytoscape (version 3.2.1). In Figure 6, node centers are colored based on the rapamycin data, with red nodes indicating a normalized F–J score of log_2_ ≥ 1, and gray nodes indicating log_2_ < 1, or no data. Node borders are colored based on selected GO Slim data (SGD GO Slim Mapper), where maroon indicates nuclear localization, and blue indicates endomembrane or vacuolar localization. Edges are colored based on the type of protein–protein data; Affinity Capture-Luminescence, Affinity Capture-MS, Affinity Capture-RNA, Affinity Capture-Western, and Reconstituted Complex are black; Two-hybrid and Protein-Fragment Complementation Assay are orange; finally, Biochemical Activity, Cocrystal Structure, Cofractionation, Colocalization, Copurification, and FRET are dotted gray.

### DNA microarrays of Rpd3L mutants

Rpd3L and Rpd3S mutants were constructed using standard methods in an EYO690 (W303) background, as described in detail previously (Worley *et al.* 2013). Overnight cultures of the EY0690 or Rpd3L mutant strains were then used to inoculate 0.75 liter of YEPD to an OD_600_ of 0.1 in a 2.8-liter conical flask, and grown shaking at 200 rpm and 30°. Once the cultures reached an OD_600_ of 0.6, 250 ml of cells were collected by vacuum filtration and frozen in liquid nitrogen. The remaining cells were then subjected to 0.375 M KCl stress for 20 min, harvested by vacuum filtration, and frozen in liquid nitrogen. Finally, the mRNA was purified from the frozen cells, converted into cDNA using reverse transcription, labeled with Cy3 or Cy5, and examined using an Agilent microarray, as described previously (Capaldi 2010; Worley *et al.* 2013).

## Results

### Automated analysis of gene expression in yeast

**Figure 1:**
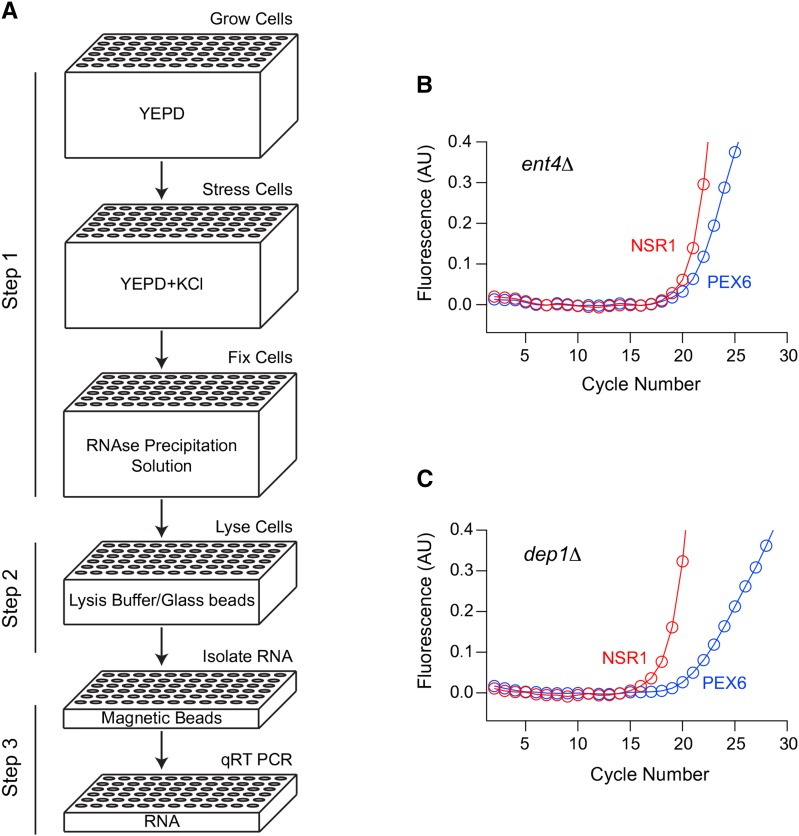
Automated analysis of gene expression in yeast. (A) Strains from the Yeast Knock Out (YKO) collection were inoculated into a 96-well plate containing YEPD medium, and grown to an OD_600_ of 0.6 in a Biomek FX robot with an integrated Liconic shaking incubator. The plates were then brought onto the deck of the robot, treated with 0.4 M KCl, rapamycin, or mock stress, and returned to the incubator. After 20 min, the plates were retrieved again but this time treated with 4 M NH_4_SO_4_ (pH 4.6) to block all further RNA synthesis and degradation. Cells were then lysed by bead-beating, and the RNA purified from each well using magnetic beads, and loaded into a PCR plate for analysis. (B and C) Duplex quantitative PCR was used to measure the expression of the Ribi gene NSR1 (FAM labeled probe; red), and the housekeeping gene PEX6 (JOE labeled probe; blue) in each well of the plate from the library. In most strains (such as *ent4*Δ from plate 1), NSR1 and PEX6 expression levels were similar. However, we also found numerous strains (such as *dep1*Δ from plate 1) with higher levels of NSR1 than PEX6. Quantitation of these data using standard procedures (see *Materials and Methods*) then led to a NSR1/PEX6 ratio for each sample (log_2_ = –2.8 for *dep1*Δ and –0.1 for *ent4*Δ).

We developed an automated pipeline and used it to measure the expression of a ribosome biogenesis gene (NSR1) in 4709 a-type strains from the yeast knock-out (YKO) collection (Winzeler *et al.* 1999; Giaever *et al.* 2002). This pipeline included three major steps (Figure 1A):

First, strains were grown to an OD_600_ of 0.6 in 96-well plates and exposed to 0.4 M KCl, 200 nM rapamycin, or mock stress. Then, at the peak of the stress response (20 min), 4 M ammonium sulfate (pH 4.6) was added to the cultures to promote protein precipitation, and block any further RNA synthesis or degradation.

Next, the 96-well plates were centrifuged to pellet the cells, and the ammonium sulfate solution was replaced with lysis buffer and glass beads. The cells were then lysed by bead-beating, and the plates centrifuged a second time to remove insoluble debris.

Finally, the RNA was purified from the lysates in each plate using silicon-coated magnetic beads, and loaded into a 96-well PCR plate. The gene expression levels in each strain were then measured using quantitative PCR—generally following expression of NSR1, and the housekeeping gene PEX6 (Figure 1, B and C).

All of the steps in the pipeline, with the exception of bead-beating and centrifugation, were performed on a Biomek FX liquid handling workstation with an integrated Liconic incubator. This ensured that all wells and plates were treated in an identical way, making it possible to compare data across strains and days (see *Materials and Methods*).

### Testing the pipeline

To test our pipeline, we grew a 96-well plate with wild-type yeast in every well, and measured NSR1 and PEX6 expression. The NSR1 and PEX6 mRNA levels were consistent across the plate, with a log_2_ standard deviation of 0.86 and 0.90, respectively (<twofold average variation). Moreover, when we normalized the NSR1 data using the PEX6 data—to account for well-to-well variation in total RNA levels—we found that the standard deviation from the mean was only 0.37 on a log_2_ scale (∼30% average variation; Figure S1).

**Figure 2:**
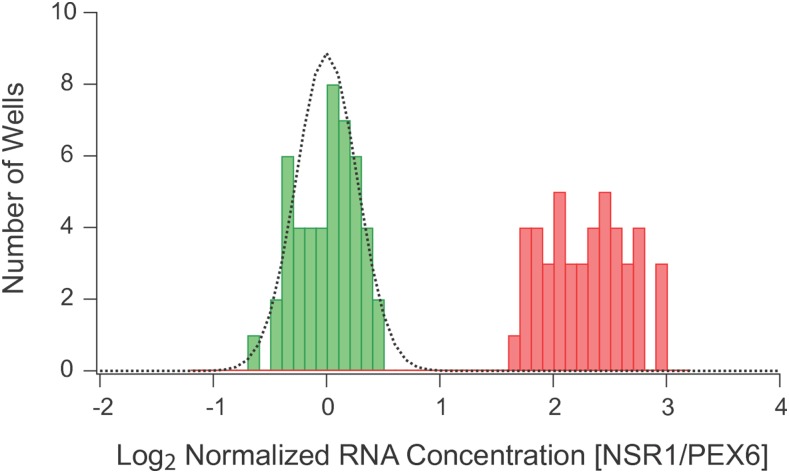
NSR1 expression levels during log growth and 0.4 M KCl stress. Histogram showing the distribution of NSR1/PEX6 expression ratios for wild-type cells grown on a single plate and then treated with 0.4 M KCl (48 samples, green) or mock stress (48 samples, red). The data were normalized (by adding a single constant to all 96 log NSR1/PEX6 ratios) so that the average signal in stress is 0.0. The dotted line shows the fit to a normal distribution with a standard deviation of 0.26 and an average of 0.0.

We then grew another plate of wild-type yeast, but this time treated half of the plate with mock stress (YEPD alone, every-other column) and the other half of the plate with 0.4 M KCl. The experiment showed that osmotic stress triggers a log_2_ = 2.3-fold average decrease in NSR1 expression (Figure 2). While this expression change is compressed compared to the log_2_ = 5-fold decrease we observe using microarray methods, the standard deviation from the mean in stress was only 0.26 on a log_2_ scale (0.36 for mock stress samples). Thus, the expression change in osmotic stress is approximately 10 times greater than the noise in our assay, indicating that our screen should be accurate enough to identify strains with moderate changes in NSR1 expression.

### Analysis of the yeast knock-out collection

After we built and tested the automated pipeline, we used it to measure the osmotic stress response in strains from the YKO library (see Methods); collecting two sets of data for 6 of the 96-well plates in the library and one set of data for the other 48 plates in the library. We then normalized the NSR1/PEX6 values to set the average expression level of the library, excluding outlier strains, to log_2_ =0.0 (see *Materials and Methods*).

**Figure 3:**
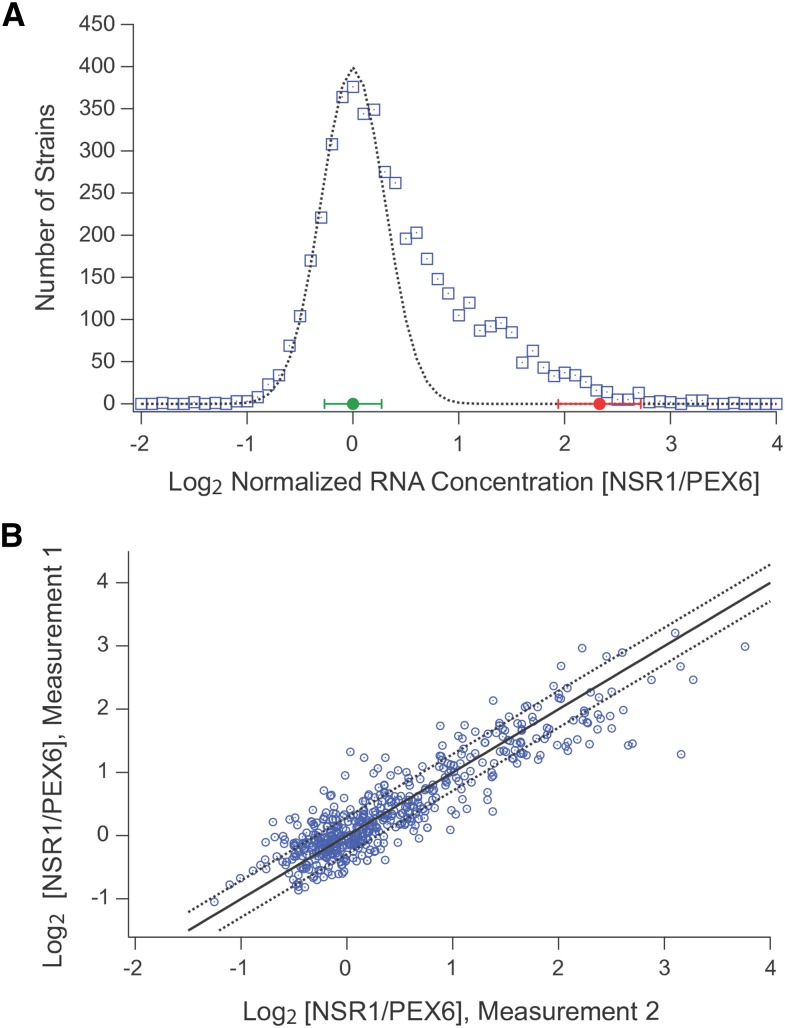
NSR1 expression levels for 4709 strains in the yeast knock-out collection. (A) Histogram showing the number of strains in the yeast knock-out library with log_2_ NSR1/PEX6 expression ratios ranging from –2 to 4 in 0.1 increment bins. All data were normalized to set the average expression ratio, minus the outliers, to 0.0 (see *Materials and Methods*). The green point and bar show the average and standard deviation of the NSR1/PEX6 ratio for the wild-type strain in stress (from Figure 3). The red point and bar shows the average and standard deviation of the NSR1/PEX6 ratio for the wild-type strain in mock stress (from Figure 3). The dotted line shows the fit to a normal distribution with an average signal of 0.0 and a standard deviation of 0.30. (B) Scatter plot showing the normalized NSR1/PEX6 expression values for 560 strains run through the automated pipeline on two separate weeks (usually more than a month apart). The solid line show the trend expected if there was a perfect correlation between datasets, the dotted line show the range expected for values that fall one standard deviation (0.3 log_2_ units) above or below this line.

Inspecting the data from the screen revealed that most of the strains in the YKO collection have a similar NSR1/PEX6 ratio, with log_2_ values ranging from –1.0 to +1.0 (Figure 3A). However, there were also over 400 outlier strains, with NSR1/PEX6 ratios ranging from log_2_ = 1.5 to 4.5 (Figure 3A).

To estimate the significance of these results, we analyzed the data from the six plates (560 strains), which were run through the pipeline twice (on separate days; Figure 3B). Overall, we found a good correlation between replicates, with a Pearson’s *r* of 0.90, and an average difference between measurements of log_2_ = 0.29. Taking this latter value as a good estimate of the average error, we then modeled the log data for the complete screen using a normal distribution with a mean of 0.0 and a standard deviation of 0.3 (Figure 3A). This model fit the data for strains with NSR1/PEX6 ratios between –1.0 and ∼0.5 very well, indicating that the variation in this range is simply due to the error in our assay. By corollary, we could then estimate the probability that a strain has a log_2_ NSR1/PEX6 ratio larger than 1.0 by chance at less than 0.1% (3.3 Z-score; Figure 3A).

Our statistical analysis suggested that there are 734 strains with a significant defect in stress dependent repression of NSR1 (log_2_ > 1.0; *P* < 0.001). However, there were two potential problems with this interpretation of the data. First, our error model is based on data from six out of 54 plates in the library, and, thus, if the error varied from plate to plate, we could be overestimating the number of strains with real defects in NSR1 repression. Second, our analysis assumes that the expression level of the housekeeping gene PEX6 is constant across all YKO collection strains, but some strains may have a higher NSR1/PEX6 ratio than expected due to a decrease in PEX6 expression.

To address these issues, we took all of the strains with a normalized NSR1/PEX6 ratio log_2_ > 1.3 (rearrayed onto six plates containing 494 strains plus 72 center peak [log_2_ = 0] controls for normalization) and ran them through our pipeline again. However, this time we measured the stress-dependent changes in the expression level of NSR1, and a different housekeeping gene: NTF2. Just over 85% of the 494 strains had log_2_ > 1.0-fold more NSR1/NTF2 than the control strains, leaving 440 strains that have significantly more NSR1 expression (*P* < 0.001) than the average strain in the YKO library in two separate assays (Table S1).

### Identification of known components in the cell growth control circuit

To estimate the false negative rate in our screen, we examined the screen data for strains missing known components in the Ribi gene control circuit. As described in the *Introduction*, TORC1, Sch9, Kcs1, Vip1, Hog1, and Rpd3L are all known to play a role in downregulating Ribi gene expression during osmotic stress. However, strains missing the TORC1 components Tor1, Kog1, Lst8 and Tco89, and the kinase Sch9 should not (and do not) show up as hits in our screen since Tor1 acts redundantly with Tor2; Tco89 has a very limited impact on TORC1 signaling; and Kog1, Lst8 and Sch9 are essential genes and thus not in the YKO library (Winzeler *et al.* 1999; Giaever *et al.* 2002; Loewith *et al.* 2002).

We did find a log_2_ = 3.1, 1.1, and 0.6 increase in NSR1 expression in the *kcs1*Δ, *vip1*Δ and *hog1*Δ strains from the YKO collection. These numbers align reasonably well with those from our previous work, where we found that deletion of Kcs1, Vip1 and Hog1 in the W303 background all caused an approximately twofold increase in Ribi gene expression in osmotic stress (Worley *et al.* 2013; Hughes Hallett *et al.* 2014). The one outlier was the *kcs1*Δ strain from the YKO library (which has a larger increase in NSR1 expression than expected), but previous work has shown that this strain behaves abnormally, and is likely carrying multiple mutations (Huang and O’Shea 2005).

We also found expression changes in YKO collection strains missing some, but not all, of the Rpd3L subunits. Previous studies have shown that Rpd3 and Pho23 are required for Ribi gene repression in stress, but little is known about the role that the other subunits in Rpd3L play in stress conditions (Alejandro-Osorio *et al.* 2009). Therefore, to build a more complete picture of Rpd3L function—and calibrate our screen—we made 14 strains, each missing one subunit of Rpd3L (Rpd3, Sin3, Ume1, Pho23, Sap30, Sds3, Cti6, Rxt2, Rxt3, Dep1, Ume6 and Ash1), or, as a control Rpd3S (Eaf3, Rco1), and measured their response to 0.4 M KCl using DNA microarrays (Carrozza *et al.* 2005a, 2005b).

**Figure 4:**
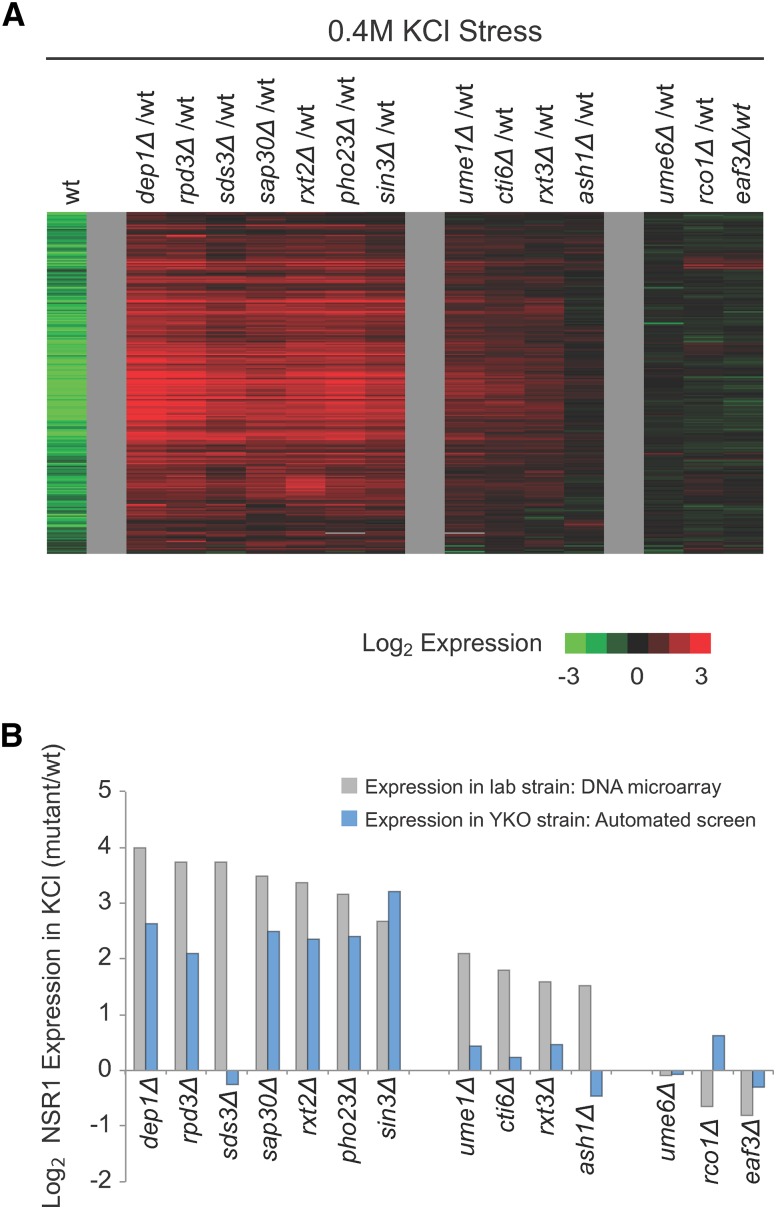
Rpd3L dependent gene expression in osmotic stress conditions. (A) DNA microarrays were use to measure the expression of Ribi genes after 20 min of 0.4 M KCl stress in the wild type strain (Column 1), and mutants missing all 14 subunits in the Rpd3L and Rpd3S complexes (Columns 2–15). In the experiment with the wild-type strain, we compared the cDNA from cells treated with stress (labeled with Cy5; red) to the cDNA from cells harvested prior to stress (labeled with Cy3; green). In experiments with the mutant strains, we compared cDNA from the mutant treated with stress (labeled with Cy5; red) to cDNA from the wild-type strain treated with stress (labeled with Cy3; green). Thus, the green bars in the first column show Ribi genes that are repressed in osmotic stress, while the red bars in each subsequent column show the genes that are hyper expressed in stress. (B) Graph showing the change in NSR1 expression caused by deletion of each subunit in Rpd3L/S as measured by DNA microarray analysis of strains made in the W303 background (gray bars) and the automated analysis of the YKO collection (blue bars).

Our microarray analysis revealed that the 14 strains missing Rpd3L or Rpd3S subunits fall into three groups (Figure 4A). The first group of strains (*rpd3*Δ, *sin3*Δ, *pho23*Δ, *dep1*Δ, *sds3*Δ, *sap30*Δ, and *rxt2*Δ) has a large defect in Ribi and RP gene repression; the second group (*ume1*Δ, *cti6*Δ, *rxt3*Δ, *ash1*Δ) has a weak to moderate defect in Ribi and RP gene repression; while the third group (*ume6*Δ, *rco1*Δ, *eaf3*Δ) has no defect in Ribi or RP gene repression.

Comparing the microarray and screen data revealed a clear trend; the screen picked up strains with large defects in NSR1 repression but not strains with small to moderate defects in NSR1 repression (Figure 4B). In fact, six out of seven gene deletions that caused a strong defect in NSR1 downregulation were identified as hits (log_2_ > 1.0) in the screen (Figure 4B). The only exception was *sds3*Δ, but in further testing we found that the inconsistency was caused by additional mutations in the strain from the YKO collection (Figure S2). In contrast, zero out of four gene deletions that caused a small to moderate defect in NSR1 downregulation in the microarray experiments were identified as hits (Figure 4B). It is therefore likely that the 440 strains with log_2_ > 1.0 more NSR1 expression during stress than the control strains includes most, if not all, of the strains in the YKO library with a strong defect in Ribi gene (NSR1) repression, but few strains with small-to-moderate defects in Ribi gene repression.

### Complexity of yeast stress and cell growth control network

**Figure 5:**
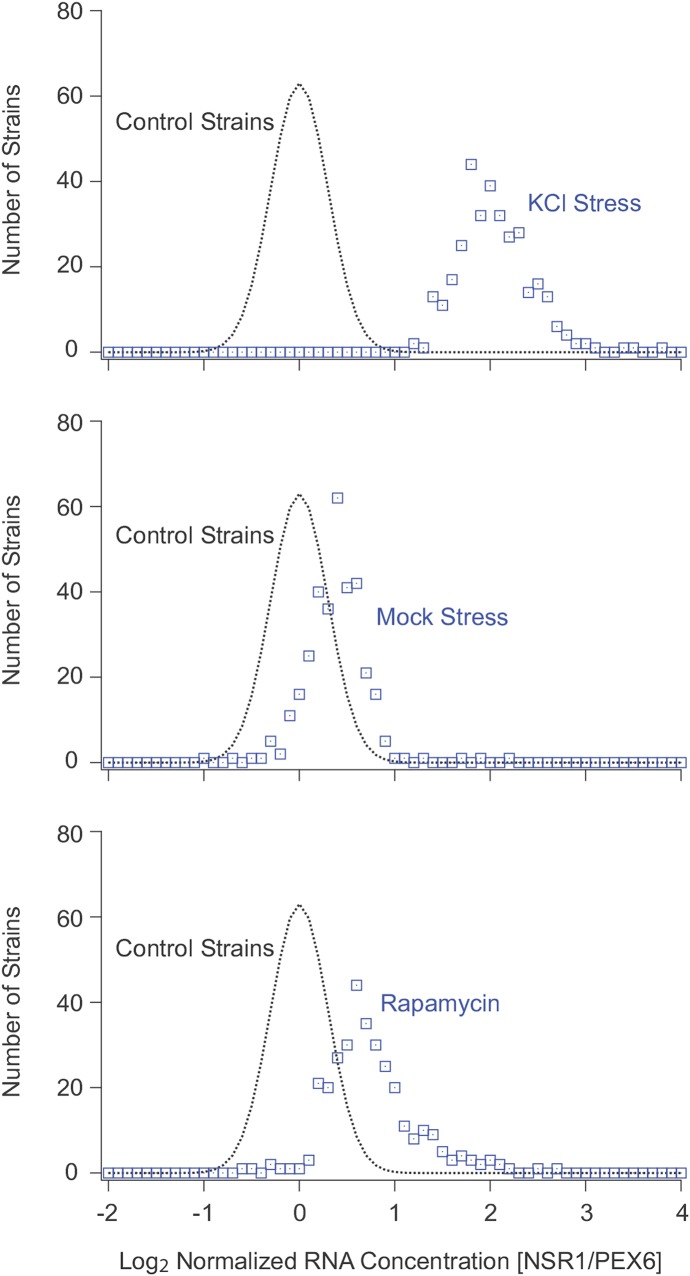
NSR1 expression levels in KCl, mock stress and rapamycin. The top 332 strains in the screen were analyzed to measure the NSR1/PEX6 ratio after 20 min in 0.4 M KCl stress (upper panel), mock stress conditions (middle panel), or 200nM rapamycin (lower panel). In all of these experiments, the 332 strains were distributed across four 96-well plates, together with 48 strains from the center of the peak in the original screen. The average NSR1/PEX6 expression level in these control strains was set to 0.0 in each experiment. Strains with defects in repressing NSR1 expression in each condition should therefore have log_2_ NSR1/PEX6 expression ratios >1.0. The dotted lines show a normal distribution with an average and standard deviation of 0.0 and 0.3 for reference.

To begin to make sense of the screen data, we set out to organize the strains with high NSR1 expression into groups. As a first step, we ran the 332 strains with NSR1 expression log_2_ > 1.4 in KCl (four plates with center peak controls for normalization; the maximum that can be processed in parallel) through our pipeline, treating them with mock stress. This experiment revealed that most of the strains with high levels of NSR1 expression in stress (top panel, Figure 5) have normal, or near normal, NSR1 expression levels during log phase growth (middle panel, Figure 5). In fact, the average NSR1/PEX6 ratio of the 332 strains in mock stress was log_2_ = 0.33, just 26% above that of the center peak control strains. Moreover, there were only five strains with log_2_ > 1.0 more NSR1 expression than the controls: *mch5*Δ (log_2_ = 2.6), *rpl16b*Δ (log_2_ = 1.8) *puf4*Δ (log_2_ = 1.7), *rpl7a*Δ (log_2_ = 1.2), and *rps7b*Δ (log_2_ = 1.1).

We then ran the 332 strains through our pipeline again, but this time treated them with the potent TORC1 inhibitor rapamycin. This experiment showed that 53 out of the 332 strains only partially downregulate NSR1 in rapamycin (normalized NSR1/PEX6 of log_2_ > 1.0), and are therefore missing genes that act downstream of TORC1 (bottom panel of Figure 5, and Table S1). Many of these 53 genes are involved in gene regulation, including 30 genes that regulate transcription [*P* < 0.001 by gene ontology (GO) analysis], and 16 genes involved in chromatin organization and biogenesis (*P* = 2e^–4^). In contrast, the 279 genes that act upstream of TORC1, or in parallel with the TORC1 pathway (log_2_ < 1.0 normalized NSR1 expression), tend to be involved in vacuolar function (30 genes, *P* = 4e^–7^) or cation homeostasis (15 genes, *P* = 5e^–4^), but not transcription (*P* = 3e^–4^ underrepresentation).

Next, to organize the hits from our screen into functional modules, we constructed a model of the Ribi gene control circuit using the physical interaction data from BioGRID (Stark *et al.* 2006). Overall, we found 1076 connections between the 440 genes/proteins with log_2_ > 1.0 NSR1 expression in salt (not including self–self interactions; see *Materials and Methods*). To test if this number of connections is significant, we also constructed 10,000 random networks, each containing 440 out of the 4709 genes studied in the screen. These networks all had less than 980 interactions (492 interactions on average), suggesting that the probability of finding 1076 connections by chance is less than 0.01%.

**Figure 6:**
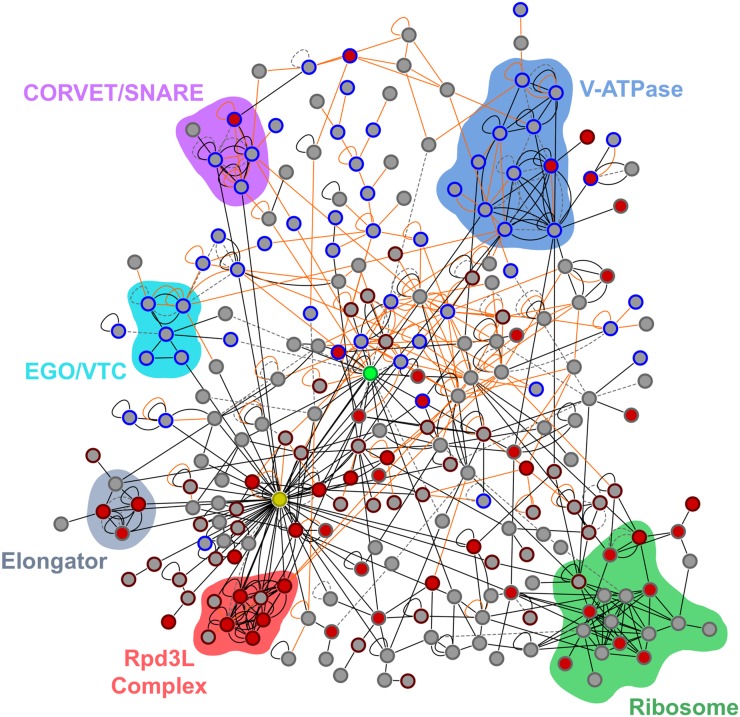
Physical interaction map for genes involved in stress-regulated growth control. The network map drawn using Cytoscape (Shannon *et al.* 2003) shows physical interactions between the 440 proteins required for robust NSR1 repression in stress, along with TORC1 for reference. Each node shows a single protein, and each edge a single physical interaction from BioGRID (Stark *et al.* 2006) colored black if it represents affinity capture or reconstituted complex data; orange if it represents two-hybrid or protein-fragment complementation data; and dotted gray if it represents FRET, biochemical activity, copurification, or other types of data. The center of each node is colored red if deletion of the protein causes a defect in rapamycin dependent downregulation of NSR1 (log_2_ > 1)—and therefore acts downstream of TORC1—and gray if it does not. Node edges are colored maroon if the protein is the nucleus, and blue if it localizes to the endomembrane system or vacuole. The green node is TORC1, and the yellow node Hht1/2. Colored regions highlight key complexes discussed in the text and listed in Table 1, Table 2, and Table 3. Only proteins with one or more physical interaction (250 in total) are shown in this figure. The highly connected protein chaperones Ssa1 and Ssb1, the RNA binding protein Slf1, and all genes that only connected to them are removed from the network for clarity. The Cytoscape file containing the full network, and all relevant information, is included in File S2.

Clustering the physical interaction data using Cytoscape (Shannon *et al.* 2003) revealed a network made up of two parts (Figure 6). The upper half includes 118 proteins connected primarily via weak or transient interactions (orange lines representing yeast two-hybrid and other weak interactions, but not IP data; Figure 6). These proteins are localized primarily to the vacuole and endomembrane system (56 blue encircled nodes; Figure 6 and Table 1) and form three distinct groups. The first group includes the A, B, C, and D subunits of the V1 portion of the vacuolar ATPase (Vma1, Vma2, Vma5, and Vma8), the c, c’, c”, and d subunits of the Vo portion of the vacuolar ATPase (Vma3, Vma11, Vma16, and Vma6), and three associated proteins (Vma21, Vma22, and Pkr1). The second group includes two components of the EGO complex [a known regulator of autophagy and TORC1 (Binda *et al.* 2009); Slm4 and Meh1], two components of the vacuolar transporter chaperone (VTC) complex, and the transporter Gap1 (EGO and VTC; Figure 6). The third group includes endosomal and vacuolar SNARE proteins (Syn8, Vam3 and Vam7), the vacuolar Rab family GTPase, Ypt7 [involved in vacuole and endosome fusion; (Schimmoller and Riezman 1993)], and a component of the CORVET membrane-tethering complex on the vacuole, Pep5. Twenty-two other genes, distributed throughout the upper portion of the network, are also involved in vesicle trafficking (bottom, Table 1), including numerous steps in transporting cargo from the ER through the Golgi and to the vacuole (Gyp5, Yip5, Emp70, Vfa1, Vab2 and Rcr1), and from the cytoplasm to vacuole (Snx4, Pfa3 and Vac8).

**Table 1:** Vacuolar, endomembrane, and vesicle trafficking genes required for the downregulation of the Ribi gene NSR1 in stress

Name	Description	Loc	[NSR1]	Down TOR	Phys Net
VMA1	Subunit A of the V1 peripheral membrane domain of V-ATPase	V	2.2	No	Yes
VMA2	Subunit B of V1 peripheral membrane domain of vacuolar H+-ATPase	V	2.3	Yes	Yes
VMA3	Proteolipid subunit c of the V0 domain of vacuolar H(+)-ATPase	V	1.9	No	Yes
VMA5	Subunit C of the V1 peripheral membrane domain of V-ATPase	V	2.2	No	Yes
VMA6	Subunit d of the V0 integral membrane domain of V-ATPase	V	2.1	No	Yes
VMA8	Subunit D of the V1 peripheral membrane domain of V-ATPase	V	2.1	No	Yes
VMA11	Vacuolar ATPase V0 domain subunit c’	V	1.5	No	Yes
VMA16	Subunit c’’ of the vacuolar ATPase	V	1.9	No	Yes
VMA21	Integral membrane protein required for V-ATPase function	ER	1.5	No	Yes
VMA22	Protein that is required for vacuolar H+-ATPase (V-ATPase) function	ER	1.9	No	Yes
PKR1	V-ATPase assembly factor	ER	1.9	No	Yes
SLM4	Component of the EGO and GSE complexes	V	3.7	No	Yes
MEH1	Component of the EGO and GSE complexes	V	1.5	No	Yes
VTC1	Subunit of the vacuolar transporter chaperone (VTC) complex	ER/V	1.4	No	Yes
VTC4	Vacuolar membrane polyphosphate polymerase	ER/V	2.3	No	Yes
GAP1	General amino acid permease	V	1.9	No	Yes
SYN8	Endosomal SNARE related to mammalian syntaxin 8	Endo	1.8	No	Yes
VAM3	Syntaxin-like vacuolar t-SNARE	V	2.6	No	Yes
VAM7	Vacuolar SNARE protein	V	2.4	No	Yes
YPT7	Rab family GTPase	V	2.5	Yes	Yes
PEP5	Histone E3 ligase, component of CORVET membrane tethering complex	V	1.9	No	Yes
RCR1	Involved in chitin deposition; may function in endosomal-vacuolar trafficking	ER	2.0	No	No
YOP1	Membrane protein that interacts with Yip1p to mediate membrane traffic	ER	1.7	No	Yes
GYP5	GTPase-activating protein (GAP) for yeast Rab family members	G	1.8	No	Yes
RGP1	Subunit of a Golgi membrane exchange factor (Ric1p-Rgp1p)	G	1.4	No	No
SYS1	Integral membrane protein of the Golgi	G	1.8	No	Yes
TVP15	Integral membrane protein; localized to late Golgi vesicles	G	1.8	No	Yes
TVP38	Integral membrane protein; localized to late Golgi vesicles	G	1.9	No	Yes
VPS52	Component of the GARP (Golgi-associated retrograde protein) complex	G	1.3	No	No
YIP5	Protein that interacts with Rab GTPases; localized to late Golgi vesicles	G	1.6	No	Yes
EMP70	Endosome-to-vacuole sorting	V	1.6	No	Yes
SNX4	Sorting nexin; involved in the retrieval of late-Golgi SNAREs	Endo	2.0	No	Yes
SNX41	Sorting nexin; involved in the retrieval of late-Golgi SNAREs	Endo	2.0	No	Yes
VFA1	Protein that interacts with Vps4p and has a role in vacuolar sorting	Endo	1.8	No	Yes
VPS5	Nexin-1 homolog; moves proteins from endosomal compartment to Golgi	Endo	1.7	No	Yes
PFA3	Palmitoyltransferase for Vac8p	V	2.4	No	Yes
VAC8	Phosphorylated and palmitoylated vacuolar membrane protein	V	2.9	No	Yes
LST4	Protein possibly involved in a post-Golgi secretory pathway		2.7	Yes	No
EDE1	Scaffold protein involved in the formation of early endocytic sites		1.6	No	Yes
ENT2	Epsin-like protein required for endocytosis and actin patch assembly		1.8	No	Yes
KIN2	Serine/threonine protein kinase involved in regulation of exocytosis		1.7	?	YES
VAB2	Subunit of the BLOC-1 complex involved in endosomal maturation		2.4	?	YES
MDR1	Cytoplasmic GTPase-activating protein; regulation of Golgi secretory function		2.4	No	No
APL4	Gamma-adaptin	Endo	1.8	No	Yes
APM1	Mu1-like medium subunit of the AP-1 complex	G	1.8	No	Yes
CHC1	Clathrin heavy chain		1.5	?	YES
DYN1	Cytoplasmic heavy chain dynein		1.7	?	YES

The top three groups of genes encode proteins highlighted in the top portion of the physical interaction (Phys Net) network shown in Figure 6; V-ATPase, EGO/VTC, and CORVET/SNARE, respectively. The fourth group lists other genes found in our screen encoding vacuolar, vesicle transport of endomembrane proteins. The third column lists the localization (Loc) of each protein. The fourth column [NSR1] lists the log_2_ NSR1/PEX6 expression ratio from the screen. The fifth column notes if the gene acts downstream of TORC1 (has log_2_ > 1 normalized NSR1/PEX6 ratio in rapamycin). The sixth column states whether the genes is part of the physical interaction network shown in Figure 6. V, vacuole; ER, endoplasmic reticulum; G, Golgi; Endo, other parts of the Endomembrane system. A question mark means that the protein/gene was not analyzed in the rapamycin subscreen.

Interestingly, almost all of the proteins in the upper portion of the Ribi gene control network, act upstream of TORC1, or in parallel with the TORC1 pathway (gray nodes in Figure 6 and Table 1). Consistent with this, TORC1 itself (green node; Figure 6) interacts with several proteins in this portion of the network (Table 2), including Vac8, a part of the CVT pathway, and Gyp5 (a GTPase-activating protein involved in ER-to-Golgi transport), and the kinases Nnk1, Fmp48 and Kdx1—forming a total of 17 interactions with proteins in the upper and lower parts of the network (Table 2).

**Table 2:** Proteins required for the downregulation of the Ribi gene NSR1 in stress that physically interact with TORC1

Name	Description	Loc	[NSR1]	Down TOR
VAC8	Vacuolar membrane protein; CVT pathway	C	2.9	Yes
GYP5	GTPase-activating protein for Rab proteins; ER to Golgi transport	C	1.8	No
DAL82	Positive regulator of allophanate inducible genes	N	2.6	No
FMP48	Protein kinase	C/M	1.7	No
KDX1	Protein kinase	M	1.5	No
NNK1	Protein kinase	C	1.8	No
SAP185	Protein that forms a complex with the Sit4p protein phosphatase	C/M	1.9	Yes
POP2	RNase of the DEDD superfamily	C	1.4	Yes
TIF1	Translation initiation factor eIF4A	C	1.6	?
MRPS17	Mitochondrial ribosomal protein of the small subunit	C	1.5	?
GAS1	Beta-1,3-glucanosyltransferase	C/M/N	1.1	?
HXT2	High-affinity glucose transporter of the major facilitator superfamily		1.9	?
ICL1	Isocitrate lyase	C	2.1	No
SAC6	Fimbrin, actin-bundling protein	C	1.8	No
TPO3	Polyamine transporter of the major facilitator superfamily	C	1.7	?
YKU80	Subunit of the telomeric Ku complex (Yku70p-Yku80p)	N	1.5	?
YLR108C	Protein of unknown function	N	1.8	Yes

The third column lists the localization (Loc) of each protein. The fourth column [NSR1] lists the log_2_ NSR1/PEX6 expression ratio from the screen. The fifth column notes if the gene/protein acts downstream of TORC1 (has log_2_ > 1 normalized NSR1/PEX6 ratio in rapamycin). A question mark means that the protein/gene was not analyzed in the rapamycin subscreen. C, cytosol; N, nucleus; M, membrane.

In the lower half of the network (also 118 genes) we find two highly connected nodes, the histone H3 proteins, Hht1/Hht2 (merged into one node for simplicity, and shown in yellow in Figure 6). Hht1/Hht2 in turn form strong interactions with three major complexes (black lines showing IP data, Figure 6). The first includes the six core subunits of Rpd3L (Rpd3, Sin3, Pho23, Sap30, Dep1, and Rxt2), as well as another Class I HDAC Hos1, and the Sin3 associated transcription factor Stb4 (Table 3). The second includes three components of the Elongator complex (part of the Pol II holoenzyme responsible for transcriptional elongation; Elp3, Elp6 and Iki3), as well as an associated kinase, Vhs1 (Table 3). The third includes 13 ribosomal proteins and four ribosome-associated proteins (Table 3).

**Table 3:** Ribosomal and nuclear genes required for the down regulation of the Ribi gene NSR1 in stress

Name	Description	Loc	[NSR1]	Down TOR	Phys Net
ELP3	Subunit of Elongator complex	N	2.8	Yes	Yes
ELP6	Subunit of Elongator complex		1.8	Yes	Yes
IKI3	Subunit of Elongator complex	N	1.8	Yes	Yes
VHS1	Cytoplasmic serine/threonine protein kinase		2.5	No	Yes
RPD3	Histone deacetylase, component of Rpd3S and Rpd3L	N	2.1	No	Yes
SIN3	Component of Rpd3S and Rpd3L	N	2.6	Yes	Yes
PHO23	Component of Rpd3L	N	2.4	Yes	Yes
SAP30	Component of Rpd3L	N	2.2	Yes	Yes
DEP1	Component of the Rpd3L	N	2.6	Yes	Yes
RXT2	Component of Rpd3L	N	2.4	Yes	Yes
HOS1	Class I histone deacetylase	N	1.9	No	Yes
STB4	Putative transcription factor	N	2.4	No	Yes
RPS6A	Protein component of the small (40S) ribosomal subunit	R	2.4	Yes	Yes
RPS7B	Protein component of the small (40S) ribosomal subunit	R	1.4	Yes	Yes
RPS9A	Protein component of the small (40S) ribosomal subunit	R	1.9	No	Yes
RPS22A	Protein component of the small (40S) ribosomal subunit	R	1.4	No	Yes
RPS17A	Protein component of the small (40S) ribosomal subunit	R	2.4	Yes	Yes
RPL2B	Ribosomal 60S subunit protein L2B	R	1.3	No	Yes
RPL6A	Ribosomal 60S subunit protein L6A	R	2.1	No	Yes
RPL6B	Ribosomal 60S subunit protein L6B	R	2.6	Yes	Yes
RPL7A	Ribosomal 60S subunit protein L7A	R	2.1	No	Yes
RPL13A	Ribosomal 60S subunit protein L13A	R	1.8	No	Yes
RPL16B	Ribosomal 60S subunit protein L16B	R	1.8	No	Yes
RPL22A	Ribosomal 60S subunit protein L22A	R	1.9	No	Yes
RPL24A	Ribosomal 60S subunit protein L24A	R	2.0	Yes	Yes
SSZ1	Hsp70 protein that interacts with Zuo1p (a DnaJ homolog)		2.0	Yes	Yes
ZUO1	Ribosome-associated chaperone	R/N	1.9	Yes	Yes
NOP12	Nucleolar protein involved in pre25S rRNA processing	N	2.1	No	Yes
RQC1	Component of the ribosome quality control complex (RQC)	R	2.0	No	Yes
RPL38	Ribosomal 60S subunit protein L38	R	2.0	Yes	No
RPL43B	Ribosomal 60S subunit protein L43B	R	1.6	No	No
RPS27A	Protein component of the small (40S) ribosomal subunit	R	2.1	No	No
CLU1	Subunit of the eukaryotic translation initiation factor 3 (eIF3)		2.3	Yes	Yes
EFT1	Elongation factor 2 (EF-2), also encoded by EFT2	R	1.6	No	Yes
TIF1	Translation initiation factor eIF4A	R	1.6	No	Yes
YGR054W	Eukaryotic initiation factor (eIF) 2A	R	2.2	No	Yes
CAF20	Phosphoprotein of the mRNA cap-binding complex		2.0	No	Yes
ASK10	Component of RNA polymerase II holoenzyme	N	2.4	No	Yes
CAF130	Subunit of the CCR4-NOT transcriptional regulatory complex		1.6	No	Yes
ELA1	Elongin A; Required for Pol II degradation	N	2.6	No	Yes
ELC1	Elongin C; Required for Pol II degradation	N	1.5	No	Yes
PGD1	Subunit of the RNA polymerase II mediator complex	N	2.0	No	Yes
NUT1	Component of the RNA polymerase II mediator complex	N	1.7	No	Yes
GIS1	Histone demethylase and transcription factor	N	1.7	No	Yes
HIR2	Subunit of HIR nucleosome assembly complex	N	2.0	No	Yes
HIR3	Subunit of the HIR complex	N	2.5	No	Yes
HPA2	Tetrameric histone acetyltransferase		1.9	No	Yes
HTA1	Histone H2A	N	2.4	Yes	Yes
IES4	Component of the INO80 chromatin remodeling complex	N	1.8	Yes	Yes
ITC1	Subunit of Isw2p-Itc1p chromatin remodeling complex	N	1.6	No	Yes
DPB4	Subunit of ISW2 chromatin accessibility complex	N	2.0	No	Yes
JHD2	JmjC domain family histone demethylase	N	2.2	Yes	Yes
RLF2	Largest subunit (p90) of the Chromatin Assembly Complex (CAF-1)	N	2.5	Yes	Yes
SAS5	Subunit of the SAS complex (Sas2p, Sas4p, Sas5p)	N	2.2	No	Yes
SWI3	Subunit of the SWI/SNF chromatin remodeling complex	N	2.5	Yes	Yes

The top three groups of genes encode proteins highlighted in the bottom portion of the physical interaction network shown in Figure 6; Elongator, Rpd3L, and Ribosome, respectively. Note that three ribosomal proteins not connected to the others by physical interactions were included in the list. The fourth group lists other genes found in our screen involved in transcription and chromatin remodeling, all of which are part of the lower half of the physical interaction network in Figure 6. The third column lists the localization (Loc) of each protein: The fourth column [NSR1] lists the log_2_ NSR1/PEX6 expression ratio from the screen. The fifth column notes if the gene acts downstream of TORC1 (has log_2_ > 1 normalized NSR1/PEX6 ratio in rapamycin). The sixth column states whether the genes is part of the physical interaction network (Phys Net) shown in Figure 6. N, nuclear; R, ribosome.

Hht1/Hht2 also interact with numerous other nuclear proteins involved in NSR1 regulation (54 maroon encircled nodes, Figure 6), including histone 2a, components of the ISW2, INO80 and SWI/SNF chromatin remodeling complexes, as well as numerous factors involved in translation and RNA decay (bottom, Table 3). Interestingly, many of the proteins in the lower half of the network, particularly those involved in chromatin remodeling and transcription, act downstream of TORC1 as per our rapamycin data (34 red nodes, Figure 6).

Outside of the portion of the Ribi gene control network connected by known physical interactions, there are many important proteins/genes (Table S1). The only enriched group includes 21 genes involved in nitrogen metabolism (*P* = 9e^–5^). However, there are also 56 enzymes in the unconnected portion of the network (including five kinases; Adk1, Bud17, Dgk1, Lsb6, and Yfh7; and five methyltransferases; Mtq2, Sam4, Trm12, Trm44, and Ymr310c), along with nine transmembrane transporters (Dip5, Hxt14, Mep1, Mup3, Pdr10, Sit1, Tom7, Ydr387c, and Yfl040w), and eight DNA binding proteins (Dal82, Hal9, Hcm1, Hop1, Sip4, Sok2, Sut2 and Znf1). These proteins may interact with components in the cell growth control network during osmotic stress—a stimulus rarely applied during large-scale studies of protein interactions and thus missing from the physical interaction network—or alter network activity by affecting the level of key metabolites in the cell.

## Discussion

We have identified 440 strains from the YKO collection that have a strong and reproducible defect in Ribi gene (NSR1) repression during osmotic stress. The proteins/genes knocked out in these strains fall into three major groups:

(1) The NSR1/Ribi regulation network contains 37 proteins involved in vesicle trafficking, 11 components of the vacuolar ATPase, and 50 other proteins that act as part of the endomembrane system (Table 1 and Table S1). These proteins probably influence NSR1 expression in a variety of ways.

Some of these proteins may directly, or indirectly, inhibit TORC1 signaling in stress. In line with this hypothesis, we found that strains missing components of the EGO complex (Meh1 and Slm4) and vacuolar ATPase–known regulators of TORC1 signaling in other conditions (Binda *et al.* 2009; Zoncu *et al.* 2011)–have large defects in NSR1 downregulation.

Other vacuole or endomembrane proteins may be important for the transport of proteins that interact with, or support the function of, TORC1 and EGO on the vacuolar membrane.

Yet other proteins in this group may be required for nutrient transport and storage, and thus deleting them could lead to changes in TORC1 and cell growth signaling. In fact, Cardenas and coworkers have already shown that disruption of the CORVET and HOPS complexes–complexes also identified in our study–cause partial inactivation of TORC1 signaling during log phase growth by inhibiting the activation of the EGO complex members Gtr1/Gtr2 (Zurita-Martinez *et al.* 2007). This constitutive TORC1 repression may then desensitize the TORC1 pathway to inhibition by osmotic stress (Figure S3).

(2) The NSR1 regulation network contains at least 24 proteins involved in chromatin silencing, six proteins involved in general transcription, and nine other DNA binding proteins (Table 3). Six of these proteins are subunits of the Class I HDAC Rpd3L—a complex that deacetylates the nucleosomes in Ribi gene promoters whenever TORC1 is inactivated (Humphrey *et al.* 2004; Huber *et al.* 2011). However, the other proteins identified in this group have not been linked to Ribi gene regulation previously. Some of these proteins probably cooperate with Rpd3L to inactivate NSR1 in stress—this is almost certainly the case for the histone H3 and H2A proteins—but others may simply regulate the transcription of critical proteins in the stress response network.

(3) The NSR1 regulation network also contains 17 ribosomal and ribosome-associated proteins, and four translation factors (Table 3). Although it is unclear how these proteins interact with the Ribi gene control network, it is well established that blocking translation using the drug cycloheximide triggers hyperactivation of TORC1 (Hara *et al.* 1998; Beugnet *et al.* 2003; Urban *et al.* 2007). It therefore seems likely that deletion of at least some of the proteins found in this group will have a similar indirect effect on TORC1 activity by inhibiting translation.

On top of the three major groups listed above, we also found three proteins known to play a role in PKA signaling (Ira2, Gpb1, and Gpr1) in our core 440 gene network, and two others (Pde1 and Pde2) that just missed the log_2_ > 1.0 cutoff (Table S1). Four of these proteins (Ira2, Gpb1, Pde1, and Pde2) are involved in limiting PKA pathway activity (Broach 2012)—suggesting that hyperactivation of the PKA pathway helps compensate for TORC1 inactivation in osmotic stress. Proteins that indirectly limit PKA pathway activity may also be part of the NSR1 regulation network.

Putting the groups of proteins listed above together with the myriad other proteins required for NSR1 repression in stress (listed in Table S1) it is clear that the Ribi, and thus the cell growth control, network is highly complex. Over seven percent of the genome (440/5820 genes) is required for proper signaling in osmotic stress conditions alone. Therefore, numerous follow up experiments will be needed to determine how such a large array of proteins contributes to the osmotic stress response. In this respect, we hope that our screen will serve as a resource that helps guide others toward key proteins and pathways in cell growth control, but remind the reader that some of our data may be misleading as many strains in the YKO collection carry mutations beyond the annotated deletion (Hughes *et al.* 2000; Teng *et al.* 2013; Giaever and Nislow 2014).

The data presented in this paper also demonstrate the power of our new method for mapping gene regulatory circuits in yeast (and potentially other organisms). It is highly quantitative, reproducible, and works well even when the resulting gene expression changes are short lived, or involve a dramatic reduction in mRNA levels. Furthermore, the method can (at least in principle) be adapted to map the regulators of any gene, simply by altering the primers/probes used in the qPCR step.

## Supplementary Material

Supporting Information
