# Supplementary material for: Genome-Wide Analysis of the TORC1 and Osmotic Stress Signaling Network in Saccharomyces cerevisiae
Source: G3 (Bethesda). 2015 Dec 16;6(2):463–74. doi: 10.1534/g3.115.025882 (PMC4751564; doi:10.1534/g3.115.025882)
Supplement: Supporting Information [file supp_6_2_463__index.html]

Genome-Wide Analysis of the TORC1 and Osmotic Stress Signaling Network in Saccharomyces cerevisiae — Supporting Information 

# Genome-Wide Analysis of the TORC1 and Osmotic Stress Signaling Network in *Saccharomyces cerevisiae*

## Supporting Information for Worley *et al.*, 2016

**Files in this Data Supplement:**

- Figure S1 - NSR1 expression levels during log growth. (.pdf, 238 KB)
- Figure S2 - Graph showing the change in NSR1/PEX6 expression caused by deletion of Sds3 in the W303 background (ACY605 strain used in microarray analysis), the BY4741 background used in the YKO collection (ACY978), and the *sds3Δ* strain from the YKO collection. (.pdf, 338 KB)
- Figure S3 - Deletion of the EGO complex components Slm4, Meh1 and Gtr1 causes constitutive repression of TORC1. (.pdf, 432 KB)
- File S1 - Supplemental Methods. (.pdf, 306 KB)
- File S2 - A Cytoscape file containing the network shown in Fig. 6. (.cys, 2,703 KB)
- Table S1 - Excel spreadsheet containing all of data from the high throughput real-time PCR screens. (.xlsx, 341 KB)
