# Supplementary material for: Genome-Wide Analysis of the TORC1 and Osmotic Stress Signaling Network in Saccharomyces cerevisiae
Source: G3 (Bethesda). 2015 Dec 16;6(2):463–74. doi: 10.1534/g3.115.025882 (PMC4751564; doi:10.1534/g3.115.025882)
Supplement: Supporting Information [file supp_g3.115.025882_FigureS1.pdf]

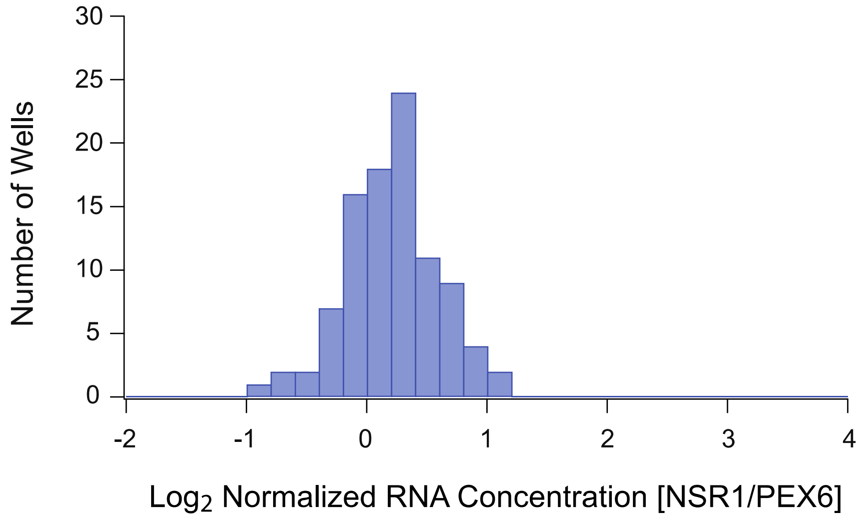

Figure S1. NSR1 expression levels during log growth. Histogram showing the distribution of NSR1/PEX6 expression ratios for wild-type cells grown on a single 96-well plate and then treated with mock stress (YEPD medium). The data was normalized (by adding a single constant to all 96 log NSR1/PEX6 ratios) so that the average signal in stress is 0.0. The standard deviation for the 96 measurements of NSR1/PEX6 expression is $\log_2 = 0.37$.
