# Supplementary material for: Genome-Wide Analysis of the TORC1 and Osmotic Stress Signaling Network in Saccharomyces cerevisiae
Source: G3 (Bethesda). 2015 Dec 16;6(2):463–74. doi: 10.1534/g3.115.025882 (PMC4751564; doi:10.1534/g3.115.025882)
Supplement: Supporting Information [file supp_g3.115.025882_FigureS2.pdf]

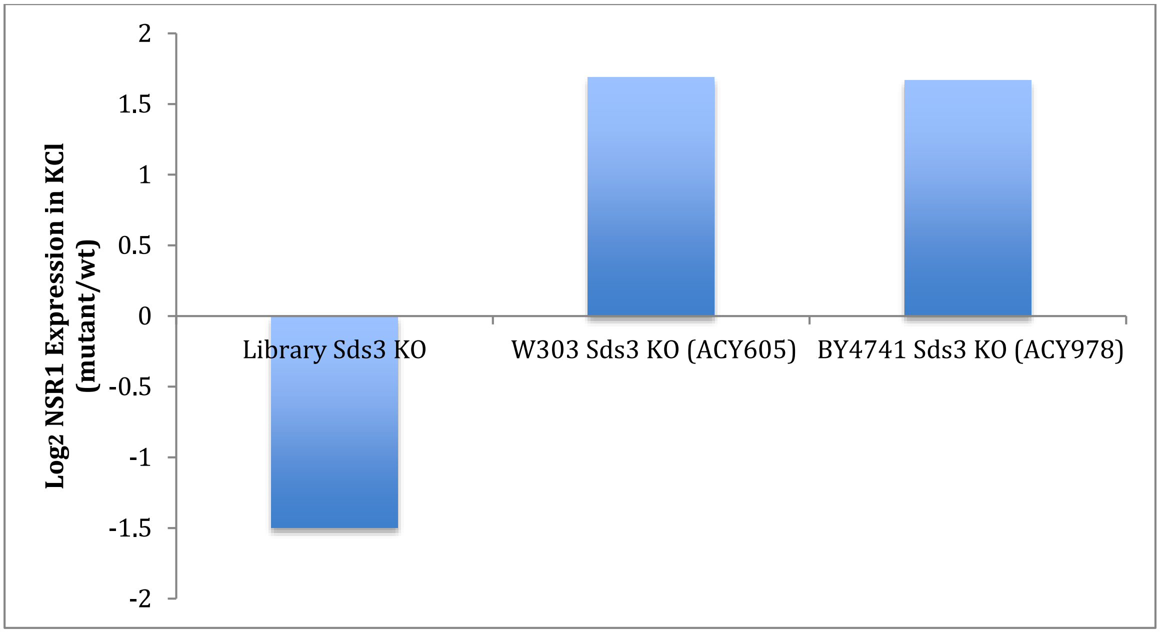

Figure S2. Graph showing the change in NSR1/PEX6 expression caused by deletion of Sds3 in the W303 background (ACY605 strain used in microarray analysis), the BY4741 background used in the YKO collection (ACY978), and the *sds3Δ* strain from the YKO collection. Each bar shows the expression level compared to that found in the wild-type strain (both 20min after treatment with 0.4M KCl), as measured by qPCR (Methods). In this experiment cells were grown and harvested as described for the DNA microarray experiments but the mRNA was purified using a RiboPure RNA purification kit (Ambion).
