# Supplementary material for: Genome-Wide Analysis of the TORC1 and Osmotic Stress Signaling Network in Saccharomyces cerevisiae
Source: G3 (Bethesda). 2015 Dec 16;6(2):463–74. doi: 10.1534/g3.115.025882 (PMC4751564; doi:10.1534/g3.115.025882)
Supplement: Supporting Information [file supp_g3.115.025882_FigureS3.pdf]

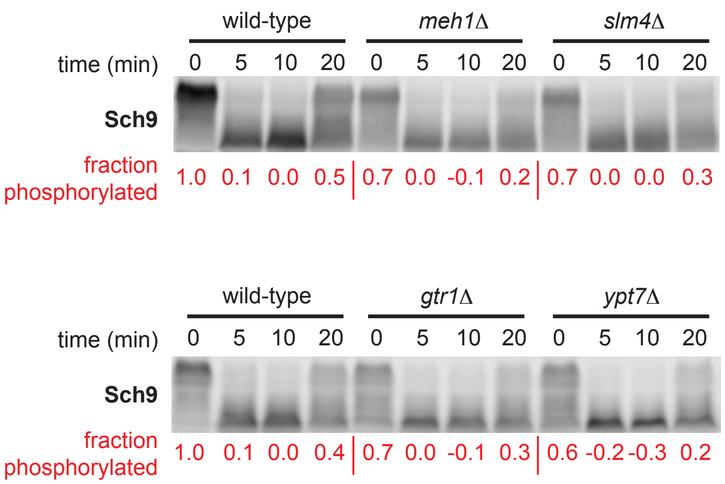

Figure S3. Deletion of the EGO complex components Slm4, Meh1 and Gtr1 causes constitutive repression of TORC1. TORC1 activity was measured by following Sch9 phosphorylation during growth in YEPD medium (time=0) and then 5, 10 and 20 min after treatment with 0.4M KCl stress, using a bandshift assay developed by Loewith and coworkers (URBAN *et al.* 2007). The protein mobility shift data was quantified using a custom MATLAB script as described in (HUGHES HALLETT *et al.* 2014) and normalized so that the wild-type values are 1.0 at time=0 min, and 0.0 at time=10 min (red numbers). The data show that deletion of Slm4, Meh1 and Gtr1 all cause a 30% decrease in Sch9 phosphorylation in YEPD medium. This may explain why the *slm4Δ*, *meh1Δ* and *gtr1Δ* strains have significant defects in the osmotic stress screen (Table S1; *gtr1Δ* just missed the $\log_2$=1.2 fold cutoff). Specifically, constitutive TORC1 repression may trigger feedback mechanisms that then limit TORC1 pathway repression in stress. However, if this is the case, the changes in the TORC1 pathway must occur at a level below TORC1, as TORC1 dependent inhibition appears to be normal during osmotic stress in all three strains (values ranging from 0.0 to -0.1 at time=10 min). We also found a constitutive TORC1 signaling defect in the strain missing Ypt7--a protein involved in vacuolar function but not thought to regulate EGO or TORC1 (BINDA *et al.* 2009). Thus, Slm4, Meh1, Gtr1 and/or Ypt7, may also block NSR1 inhibition in stress by disrupting vacuolar function (see Discussion). Note that other gene deletions, and mutations that lock Gtr1/2 in their active state, do not influence TORC1 signaling in osmotic stress (HUGHES HALLETT *et al.* 2014).
