# Supplementary material for: Genome-Wide Analysis of the TORC1 and Osmotic Stress Signaling Network in Saccharomyces cerevisiae
Source: G3 (Bethesda). 2015 Dec 16;6(2):463–74. doi: 10.1534/g3.115.025882 (PMC4751564; doi:10.1534/g3.115.025882)
Supplement: Supporting Information [file supp_g3.115.025882_FileS1.pdf]

File S1. SUPPLEMENTAL METHODS

Program I (Grow, Stress, and Harvest)

The operator loads 1-8 sets of barcoded plates into the Biomek. Each set includes: one overnight culture plate, one 2.2 ml square well plate with 550ul of YEPD and a sterile 3.2mm stainless steel mixing bead in each well (media plate), two 2.2 ml square well plates containing 850ul of RI buffer in each well (RI buffer plates), one 2.2ml square well plate containing 1.875M KCl or 1ug/ml rapamycin in YEPD in each well (stress plate; volume depends on the number of plates that are being processed), and one 300ul optical read plate. The media and stress plates are then transferred into the Liconic incubator (shaking at 1200rpm and 30C) while the RI buffer plates, overnight plate, and a set of P50 and P200 aerosol resistant tips are transferred into the Cytomat. Then, when the operator activates the inoculation routine, a barcode matched overnight culture, media, and optical read plate are moved to the deck of the robot (the media plate is loaded onto a peltier position on the deck and held at 30C). The yeast in the overnight culture plate is then resuspended by pipetting 39ul up and down 6 times, and 6ul of culture is transferred into the media plate and mixed by pipetting 39ul up and down six more times. The media plate is then moved from the peltier position to an orbital shaker, vortexed at 900rpm for 20s, and moved back to the peltier position. 250ul of culture is then transferred into the optical read plate (using two steps with P200 tips) and the OD_{600} read by the operator. Once the OD is read the cultures are transferred back from the optical read plate into the media plate and all of the plates returned to the incubator. The last transfer (from the optical read plate to media plate) uses a custom pipetting template to ensure that yeast are removed from the edge of each well. Specifically, the transfer volumes were split in 40 aspirations, each of which is shifted 10 degrees around the perimeter of the well. The inoculation steps are then repeated for up to seven additional sets of plates (at intervals chosen by the operator, usually 1hr).

During cell growth, the operator is able to read the OD of any plate in the robot. Here again, the operator activates a routine where the media plate is moved to a peltier position on the deck of the robot (held at 30C), 250ul of culture is transferred to the optical read plate, the OD_{600} read, the cultures transferred back to the media plate, and then all plates are moved back into the Liconic incubator. In general, the OD_{600} was read 2-3 times after the initial inoculation, so that we could accurately predict the time-point at which cells reach an OD_{600} of 0.6.

Once the cells reached an OD_{600} of 0.6, the stress treatment routine is activated. First, the media plate and salt stress (or rapamycin) plates are retrieved from the incubator and the RI buffer plates and P200 tips are retrieved from the Cytomat. Then, 150ul of culture is transferred from the media plate to the first RI buffer plate, and mixed by aspirating 150ul from 1mm above the bottom of the plate and dispensing it again at 15mm above the bottom of the plate, at 100ul/s for 2 min. 100ul of YEPD containing KCl or rapamycin is then transferred from the stress plate

to the media plate using a moving dispense step, starting at the bottom of the plate and ascending to 14.5mm above the bottom at 75ul/s, followed by three 25ul mixes at 100ul/s, and the samples are shaken at 900rpm for 10s and returned to the incubator. Approximately 19min later the media plate is moved back to peltier position on the deck and 150ul of culture transferred to the second RI buffer plate (as described above). The plates containing cells in RI buffer were then stored at -20C.

Program II (Aspirate RI Buffer and resuspend cells in Lysis Buffer)

After the yeast in RI buffer are thawed by centrifugation they are loaded into the Biomek along with a plate containing 400ul lysis buffer and 300ul zirconia beads. Then, the RI buffer is removed from each plate, 150ul at a time (using a P200 tip). During this aspiration step the tip is placed 1mm above the bottom of the well, off center by 80% of the well diameter, and the liquid removed at 20ul/s to ensure that the cell pellet remains intact. RI buffer is then dispensed into a position on the deck loaded with a plate shaped funnel made using a 3D printer (that transfers the liquid to a beaker so that a reservoir does not have to be emptied multiple times during the program). Once the RI buffer is removed, the robot pauses and waits for a prompt from the user (to make sure they are ready to move the plate to the bead beater). Once activated, the robot then transfers 400ul of lysis buffer to the plates containing the cell pellet, moves the plate to the orbital shaker on the deck of the robot, and then performs a mixing loop four times. This mixing loop involves aspirating 150ul from 0.8mm above the bottom of the well at 90ul/s and dispensing the buffer at 2mm above the bottom of the well at 90ul/s, 3 times per loop, and then shakes the plate at 1000rpm for 10s. At the end of the mixing procedure, 390ul of yeast in lysis buffer is transferred into the lysis plate containing glass beads, and immediately loaded into the bead beater by the operator. Once bead-beating is complete, the plates were centrifuged at 4C and then loaded back into the robot for RNA purification.

Program III (RNA Purification)

The operator loads 1-8 sets of barcoded plates into the Biomek (4 sets is the most that can processed in a single 12hr day) along with aerosol resistant P200 and P50 tips. Each set includes: the cell lysate plate, an empty RNA plate (rigid, full skirt, PCR plate), an empty binding reaction plate (rigid, full skirt, PCR plate), and an empty 2.2ml well waste plate. The lysate plates are then moved into the Liconic incubator (at 4C) and the remaining sets of plates and tips are moved into the Cytomat. The user then loads a reservoir containing elution buffer onto the peltier position, and reservoirs containing wash buffer 1, wash buffer 2, RNA rebinding buffer (1.5x wash buffer 1), a 700ul plate containing Turbo DNAse, a rigid PCR plate with MagMAX RNA isolation beads, and a magnetic stand (Ambion #AM10027) onto other positions on the deck. The Turbo DNAase was then moved to the Liconic incubator (at 4C). Note that we used a 3D printer to make a 1.8mm plate that sat over the pins

on the magnetic stand. This lowered the position of the beads on the side of the tube so that we could elute the RNA in a lower volume.

Once the plates and reservoirs are loaded, the first lysate plate is moved to the deck, and 100ul of cell lysate is moved to the binding reaction plate using P200 tips. To avoid pulling glass beads into the tips the aspiration was done in 12 steps, each step involves: Aspirating 0.1x of the volume at 11.4 mm above the bottom of the well (at the level of the top of the beads) at 60 ul/s, pausing in place for 0.5s, and dispensing 0.02x of the volume at 80ul/s. After 12 cycles the robot aspirates 0.04X of the volume and then dispenses the liquid into the binding plate at 1mm above the bottom of the plate. 150ul of isopropanol is then added to the binding plate, and mixed for 1 minute by aspirating 150ul at 0.8mm above the bottom of the well at 80ul/s and dispensing 150ul at 3.5mm above the bottom of the well at 150ul/s. The robot then resuspends the magMAX binding beads by performing the following sequence 20 times using p50 tips:

- Aspirate 150ul at 1.2mm above the bottom of the well at 80ul/s
- Move the tip to 10mm above the bottom of the well on center
- **Move the tip to 1.2mm off center (the side of the well) at 45 degrees (2 o'clock) and 10mm above the bottom of the well.**
- (1) Dispense the volume at 150ul/s
- (2) Move the tip to 10mm above the bottom of the well on center
- (3) Aspirate 30ul at 1.2mm above the bottom of the well at 80ul/s
- (4) Move the tip to 10mm above the bottom of the well on center
- **Move the tip to 1.2mm off center at 135 degrees (10 o'clock) and 10mm above the bottom of the well.**
- Repeat steps 1-4 from above
- **Move the tip to 1.2mm off center at 225 degrees (8 o'clock) and 10mm above the bottom of the well.**
- Repeat steps 1-4 from above
- **Move the tip to 1.2mm off center at 315 degrees (5 o'clock) and 10mm above the bottom of the well.**
- Repeat steps 1-4 from above
- Dispense the volume at 150ul/s

The robot then transfers 20ul of the MagMAX bead slurry to each well in the binding plate. The robot then uses P200 tips to mix the isopropanol, lysate and beads for 7min. The pipetting sequence used is:

- Aspirate 150ul at 0.8mm above the bottom of the well at 80ul/s
- Move the tip to 10mm above the bottom of the well on center
- **Move the tip to 1.2mm off center (the side of the well) at 45 degrees (2 o'clock) and 10mm above the bottom of the well.**
- (1) Dispense the volume at 150ul/sec

- (2) Move the tip to 10mm above the bottom of the well on center
- (3) Aspirate 150ul at 0.8mm above the bottom of the well at 80ul/sec
- (4) Move the tip to 10mm above the bottom of the well on center
- **Move the tip to 1.2mm off center at 135 degrees (10 o'clock) and 10mm above the bottom of the well.**
- Repeat steps 1-4 from above
- **Move the tip to 1.2mm off center at 225 degrees (8 o'clock) and 10mm above the bottom of the well.**
- Repeat steps 1-4 from above
- **Move the tip to 1.2mm off center at 315 degrees (5 o'clock) and 10mm above the bottom of the well.**
- Repeat steps 1-4 from above
- Dispense the volume at 150ul/s
- Move the tip to 10 mm above the bottom of the well on center

The robot then moves the binding plate to the magnetic stand and pauses for 4min. The robot then moves the supernatant to the waste plate using the following pipetting steps and P200 tips:

- Aspirate 75ul at 0.9mm above the bottom of the well at 10ul/s
- Dispense 75ul at 1.2mm below the top of the well at 75% off center, 90 degrees (12 o'clock)
- Aspirate 75ul at 0.9mm above the bottom of the well at 10ul/s
- Dispense 75ul at 1.2mm below the top of the well at 75% off center, 90 degrees (12 o'clock)
- Aspirate 50ul in four parts at 20ul/s
  - 12.5ul while descending from 1mm above the bottom of the well to -0.1mm above the bottom of the well (to be sure to push on the bottom of the well)
  - Pause 50 ms
  - 12.5ul while ascending from -0.1mm above the bottom of the well to 1mm above the bottom of the well
  - Pause 50ms
  - 12.5ul while ascending from 1mm above the bottom of the well to -0.1mm above the bottom of the well
  - Pause 50ms
  - 12.5ul while ascending from -0.1mm above the bottom to 1mm above the bottom
  - Pause 50ms

- Dispense 50ul at 1.2mm below the top of the well at 75% off center, 90 degrees (12 o'clock)

Next, the robot removes the binding reaction plate from the magnetic stand and transfers 150ul of wash buffer 1 to the binding plate using a P200 tip and mixes the beads and buffer for 2min. During this, and other wash steps, we found that it is critical to dispense buffer at different points around the tube. In prototype versions of the protocol missing this step we got highly variable data due to beads sticking to the sides of some tubes. The pipetting sequence we used is:

- Aspirate 115ul at 0.8mm above the bottom of the well at 80ul/s
- Move the tip to 10mm above the bottom of the well on center
- **Move the tip to 1.2mm off center (the side of the well) at 45 degrees (2 o'clock) and 10mm above the bottom of the well.**
- (1) Dispense the volume at 150ul/s
- (2) Move the tip to 10mm above the bottom of the well on center
- (3) Aspirate 115ul at 0.8mm above the bottom of the well at 80ul/s
- (4) Move the tip to 10mm above the bottom of the well on center
- **Move the tip to 1.2mm off center at 135 degrees (10 o'clock) and 10mm above the bottom of the well.**
- Repeat steps 1-4 from above
- **Move the tip to 1.2mm off center at 225 degrees (8 o'clock) and 10mm above the bottom of the well.**
- Repeat steps 1-4 from above
- **Move the tip to 1.2 mm off center at 315 degrees (5 o'clock) and 10mm above the bottom of the well.**
- Repeat steps 1-4 from above
- Dispense the volume at 150ul/s
- Move the tip to 10mm above the bottom of the well on center

The robot then moves the binding reaction plate to the magnetic stand again, pauses for 2min, and then transfers the supernatant to the waste plate using the following pipetting steps and P200 tips:

- Aspirate 75ul at 0.9mm above the bottom of the well at 10ul/s
- Dispense 75ul at 1.2mm below the top of the well at 75% off center, 90 degrees (12 o'clock)
- Aspirate 75ul at 0.9mm above the bottom of the well at 10ul/s
- Dispense 75ul at 1.2mm below the top of the well at 75% off center, 90 degrees (12 o'clock)
- Aspirate 50ul in four parts at 20ul/s (as defined above)

- Dispense 50ul at 1.2mm below the top of the well at 75% off center, 90 degrees (12 o'clock)

The robot then removes the binding plate from the magnetic stand again and repeats the procedures described above, but this time using wash buffer 2. Once the washing procedures are complete, the Turbo DNase plate is brought out onto the deck of the robot and 50ul of Turbo Dnase in buffer is added to each well. The robot then mixes the beads and Dnase for 25min. The pipetting sequence is:

- Aspirate 50ul at 1mm above the bottom of the well at 80ul/s
- Move the tip to 10mm above the bottom of the well on center
- **Move the tip to 1.2mm off center (the side of the well) at 45 degrees (2 o'clock) and 10mm above the bottom of the well.**
- (1) Dispense the volume at 150ul/s
- (2) Move the tip to 10mm above the bottom of the well on center
- (3) Aspirate 50ul at 1 mm above the bottom of the well at 80ul/s
- (4) Move the tip to 10mm above the bottom of the well on center
- **Move the tip to 1.2mm off center at 135 degrees (10 o'clock) and 10mm above the bottom of the well.**
- Repeat steps 1-4 from above
- **Move the tip to 1.2mm off center at 225 degrees (8 o'clock) and 10mm above the bottom of the well.**
- Repeat steps 1-4 from above
- **Move the tip to 1.2mm off center at 315 degrees (5 o'clock) and 10mm above the bottom of the well.**
- Repeat steps 1-4 from above
- Dispense the volume at 150ul/s
- Move the tip to 10mm above the bottom of the well on center

The robot then adds 100ul of RNA rebind solution to the reaction plate, and mixes the solution for 5min. The pipetting sequence is:

- Aspirate 150ul at 1 mm above the bottom of the well at 80ul/s
- Move the tip to 10mm above the bottom of the well on center
- **Move the tip to 1.2mm off center at 45 degrees (2 o'clock) and 10mm above the bottom of the well.**
- (1) Dispense the volume at 150ul/s
- (2) Move the tip to 10mm above the bottom of the well on center
- (3) Aspirate 150ul at 1mm above the bottom of the well at 80ul/s
- (4) Move the tip to 10mm above the bottom of the well on center
- **Move the tip to 1.2mm off center at 135 degrees (10 o'clock) and 10mm above the bottom of the well.**

- Repeat steps 1-4 from above
- **Move the tip to 1.2 mm off center at 225 degrees (8 o'clock) and 10 mm above the bottom of the well.**
- Repeat steps 1-4 from above
- **Move the tip to 1.2mm off center at 315 degrees (5 o'clock) and 10mm above the bottom of the well.**
- Repeat steps 1-4 from above
- Dispense the volume at 150ul/s
- Move the tip to 10mm above the bottom of the well on center

The robot then moves the binding reaction plate to the magnetic stand and pauses for 2min before carrying out an additional round of washing with wash buffer 2. Once the final washes are complete 30ul of Elution Buffer (heated to 55C on the peltier position) is added to the beads in the binding plate (off the magnet) using P50 tips, at a position 5.8mm above the bottom of the well. The robot then mixes the beads and elution buffer using the following pipetting sequence:

- Aspirate 30ul at 1mm above the bottom of the well at 80ul/s
- Move the tip to 10mm above the bottom of the well on center
- **Move the tip to 1.2mm off center (the side of the well) at 45 degrees (2 o'clock) and 10mm above the bottom of the well.**
- (1) Dispense the volume at 150ul/sec
- (2) Move the tip to 10mm above the bottom of the well on center
- (3) Aspirate 30ul at 1 mm above the bottom of the well at 80ul/s
- (4) Move the tip to 10 mm above the bottom of the well on center
- **Move the tip to 1.2mm off center at 135 degrees (10 o'clock) and 10mm above the bottom of the well.**
- Repeat steps 1-4 from above
- **Move the tip to 1.2mm off center at 225 degrees (8 o'clock) and 10mm above the bottom of the well.**
- Repeat steps 1-4 from above
- **Move the tip to 1.2mm off center at 315 degrees (5 o'clock) and 10mm above the bottom of the well.**
- Repeat steps 1-4 from above
- Dispense the volume at 150ul/s
- Move the tip to 10mm above the bottom of the well on center

The robot then moves the binding reaction plate to the magnetic stand, and pauses for 2min, while it retrieves the RNA plate from the Cytomat. 30ul of eluate is then transferred from the binding plate to the RNA plate using a P50 tip, using both a 30ul pipetting step and a 10ul pipetting step (at 0.9mm above the bottom of the well) to ensure all liquid is transferred. The robot then transfers the RNA plate to

the Liconic incubator (at 4C) and moves to the next plate in the sequence. The plates containing RNA were stored at -80C and later analyzed by qPCR.
